# Synchronization of follicular wave emergence does not improve embryonic yield in superovulated ewes

**DOI:** 10.1590/1984-3143-AR2021-0084

**Published:** 2022-01-07

**Authors:** Oscar Oliveira Brasil, Nathalia Hack Moreira, Fábia Fernanda Cardoso de Barros da Conceição, Paula Lorena Grangeira Souto, Cleidson Manoel Gomes da Silva, Alexandre Floriani Ramos

**Affiliations:** 1 Departamento de Agronomia e Medicina Veterinária Universidade de Brasília Brasília DF Brasil Departamento de Agronomia e Medicina Veterinária, Universidade de Brasília, Brasília, DF, Brasil; 2 Departamento de Medicina Veterinária Faculdades Integradas da União Educacional do Planalto Central Gama DF Brasil Departamento de Medicina Veterinária, Faculdades Integradas da União Educacional do Planalto Central, Gama, DF, Brasil; 3 Instituto de Estudos do Trópico Úmido Universidade Federal do Sul e Sudeste do Pará Xinguara PA Brasil Instituto de Estudos do Trópico Úmido, Universidade Federal do Sul e Sudeste do Pará, Xinguara, PA, Brasil; 4 Departamento de Conservação de Recursos Genéticos Animais Embrapa Recursos Genéticos e Biotecnologia Brasília DF Brasil Departamento de Conservação de Recursos Genéticos Animais, Embrapa Recursos Genéticos e Biotecnologia, Brasília, DF, Brasil

**Keywords:** embryos, estradiol-17β, recruitment, follicle, superovulation

## Abstract

The present study aimed to investigate the effects of a combination of progesterone with different doses of E-17β on following end points: (1) ovarian follicular dynamics and emergence of a new follicular wave, and (2) superovulatory response and embryo yield. In Experiment 1, 28 ewes were randomly divided into four groups (*n* = 7) to receive either 2.0 mg, 1.0 mg, 0.5 mg or none E-17β one day after insertion of a progesterone device. The different doses of estradiol similarly delayed the moment of follicular emergence (overall mean = 3.1 ± 1.0 days vs. control group = 0.86 ± 1.0 days; P < 0.01), but the emergence of the new wave showed greater synchronization with the 0.5 mg dosage of E-17β. In Experiment 2, sixty-two donor ewes received an internal progesterone release device (day -1) for 7 d and 1 d after the insertion of this device (day 0) were allocated randomly to receive 0.5 mg of E-17β or only the vehicle (control group). Superstimulation was initiated on day 3 with the administration of 133 mg of pFSH in eight decreasing doses. Contrary to expectations, the protocol with the administration of 0.5 mg E-17β did not improve the percentage of donors with > 2 CL, the number of CL and the production of embryos (P > 0.05). It was concluded that the combination of progesterone and 0.5 mg E-17β was more efficient in synchronizing the emergence of the new follicular wave, however this approach seems to be unnecessary in ewe’s superovulation programs.

## Introduction

Multiple ovulation and embryo transfer (MOET) technologies are applied in sheep in order to increase the progeny from selected ewes. The superovulation protocol typically entails 14 d of progesterone administration, with FSH treatment beginning approximately 3 d before the removal of the progesterone-releasing device (Cognie et al., 2003; Lagares et al., 2021), i.e., at random stages of follicular wave development. However, an important limiting factor still affecting the success of MOET programs is the variability of the ovarian response and embryo yield (reviewed by Menchaca et al., 2010 and Bartlewski et al., 2016). This variation is caused by extrinsic and intrinsic factors such as source and purity of hormones, administration protocols, breed, age, nutritional and reproductive status (González-Bulnes et al., 2004). Furthermore, differences in follicular wave status at the beginning of ovarian superstimulation treatments appear to be one of the major sources of variability (Rubianes et al., 1995; González-Bulnes et al., 2000; González-Bulnes et al., 2002; Veiga-Lopez et al., 2005; Bartlewski et al., 2008b), limiting the use of superovulation in commercial and research applications.

Studies of the influence of ovarian follicular status at the beginning of ovarian superstimulation treatment showed that ovulatory response and total number of transferable embryos are positively affected by the number of small follicles (González-Bulnes et al., 2000; Bartlewski et al., 2008a) and negatively affected by the presence of a large follicle (González-Bulnes et al., 2002; Veiga-Lopez et al., 2005) on the first day of the ovarian superstimulation regimen. To obviate these problems, an alternative approach is to initiate superstimulation treatments subsequent to the exogenous control of follicular wave emergence (Bo et al., 2002; Menchaca et al., 2010).

Follicular wave synchronization has become customary in cattle undergoing superovulation (Mapletoft et al., 2002). Progesterone and estradiol (E2) in combination significantly reduce the variability associated with traditional superovulation treatments in cows (Bo et al., 2002; Mapletoft et al., 2002). It is expected that the same would occur in sheep but this has not been studied in depth to the same extent as in cattle.

Two experiments were conducted to evaluate the follicular dynamics and embryo yield in cyclic hair sheep pretreated with progesterone and E-17β in combination. The objective of the first experiment was to evaluate the effect of progesterone combined with different dosages of E-17β on ovarian follicular dynamics and synchrony of new follicular wave emergence. Posteriorly, the second experiment aimed to evaluate ovulatory response and embryo yield when ovarian superstimulation is started at the beginning of the follicular wave.

## Material and methods

### Experimental location and animals

This study was conducted at Embrapa Genetic Resources and Biotechnology located in Brasília (Mid-West region of Brazil) at 15°88’ south latitude and 48°01’ west longitude, altitude ranging from 1050 to 1250 m above sea level. This region has a tropical savanna climate, with dry winters and rainy summers, Type AW by Köppen classification (Alvares et al., 2013). Ethical concerns were taken into account by adhering to local animal welfare regulations and practices. All the procedures of this study were approved by the Animal Ethics Committee of the Embrapa Genetic Resources and Biotechnology (Protocol CEUA-Cenargen 721/2015).

Ninety ewes sexually mature, clinically healthy, cyclic Santa Inês breed between 2 to 5 years old and with a body condition score ≥ 2.5 (range 1 to 5) were used in all of the experiments. One week prior to the start of each experiment, a preliminary evaluation was performed via transrectal ultrasonography in order to select those ewes that had one or more corpora lutea.

### Ultrasound technique

Transrectal ultrasonographic examination of ovaries was performed with high-resolution, real-time B-mode ultrasound equipment (DP-2200Vet; Shenzhen Mindray Bio-Medical Electronics Co. Ltd., Nanshan, Shenzhen, P.R. China) equipped with a stiffened 7.5 MHz, linear-array multifrequency transducer. Ewes underwent ultrasound examination while restrained in a holding crate in a standing position. This technique has been validated for monitoring ovarian follicular dynamics and CL detection in sheep (Viñoles et al., 2004). One experienced operator performed all the examinations. Ovarian follicles and CL were measured using internal electronic calipers, and the number, diameter and topographic location of CL and all antral follicles ≥ 1mm were sketched on ovarian charts.

### Experiment 1

During April-May, twenty-eight cyclic hair sheep (average body weight 45 ± 7 kg), were used in this experiment. The animals were kept in a pasture of Tifton (Cynodon spp.), from 08:00 to 16:45 h, and after this period in collective stalls with Tifton hay *ad libitum*. Throughout the period, the animals had unlimited access to water and mineral salt. The estrus cycle of all ewes was synchronized via the insertion (day -1) of a progesterone controlled-internal-drug-release device (Eazi-Breed^™^ CIDR®-Controlled Internal Drug Release, InterAg, Hamilton, New Zealand). On day 0, the ewes were randomly divided into four groups (n = 7) which received 2.0 mg, 1.0 mg, 0.5 mg of E-17β i.m. (Sigma-Aldrich, St. Louis, MO, USA) dissolved in 1 mL of sesame oil (Sigma-Aldrich, St. Louis, MO, USA) or only 1 mL of sesame oil (Control group). The CIDR was kept in place until the ascertaining, by means of transrectal ultrasound, of the onset of regression of the largest follicle of the new follicular wave that emerged after the insertion of CIDR. Because of the time required for the ultrasonographic evaluation of follicular dynamics, this experiment was carried out in two moments with an equal number of animals from each group, the first period with 16 and the last 12 animals.

Ultrasonography evaluations were performed at the time of CIDR insertion, 24 h later and then every 12 h until the onset of the atresia of the dominant follicles of the new wave. Follicular data (follicles diameter ≥ 3 mm) were combined for both ovaries. Interpretation of follicular dynamics was adapted using the criteria reported by Bartlewski et al. (1999). A follicular wave consisted of a follicle or a group of follicles that emerged from 3 mm in diameter and grew to ≥ 4 mm before regression. The day that the biggest follicle in the wave was first detected at 3 mm was the day of wave emergence. If more than one follicle attained the same maximum size, the follicle that reached the maximum diameter first was regarded as the largest follicle of the wave.

The following characteristics of follicular waves were determined for each ewe: (1) diameter of the largest follicle on day -1, i.e., at CIDR insert; (2) diameter of largest follicle on day 0, i.e., at E-17β administration; (3) CL diameter on day 0; (4) number of days for follicular wave emergence; (5) diameter of largest follicle of new wave; (6) duration of follicle growth and static phases as well as wave length; and (7) growth rate of largest follicle. The growth phase was defined as the time taken by the follicle to grow from 3 mm to its maximum size; the growth rate was calculated by dividing follicular growth (maximum diameter - 3 mm) by the growth phase; the static phase was the interval between the end of the growth phase and the beginning of follicular regression; and the wave length was defined as the interval between the end of the static phase and the emergence of the wave. During the static phase, when follicular reduction was observed in two consecutive ultrasonography evaluations, the time of the first evaluation which showed reduction was determined to be the onset of follicular regression.

### Experiment 2

**Figure 1:**
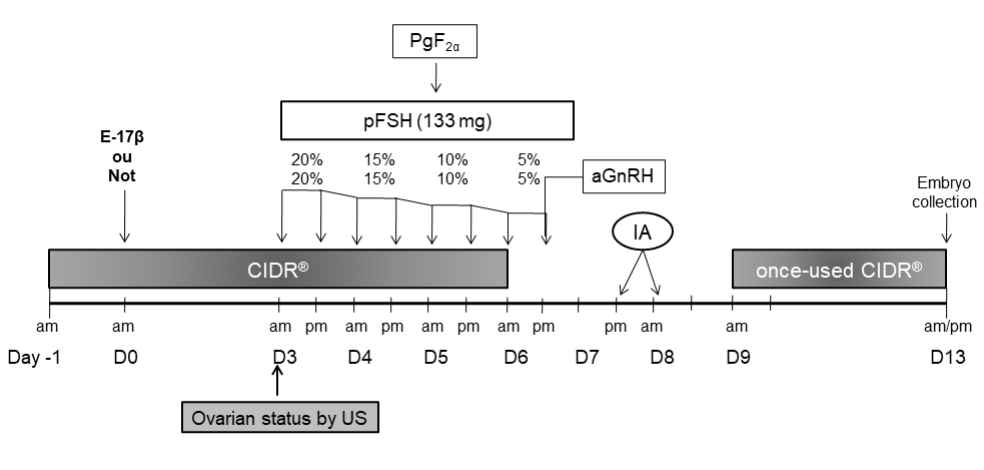
Treatment schedule for cyclic hair sheep receiving a CIDR (day -1) for 7 d. Donor’s ewes were randomly separated into two experimental groups, with one group receiving one dose of E-17β at 24 h after CIDR insertion. All ewes received two injections of FSH i.m. per day over 4 d starting on day 3. Transrectal ultrasonography was performed on day 3 (n = 12). All ewes were inseminated 36 and 48 h after CIDR withdrawal and the previously used CIDR was reinserted 1 d after the second insemination (day 9). Ova/embryos were surgically collected 5 d after the second insemination (day 13).

During October, sixty-two cyclic hair sheep (average body weight 39 ± 5 kg), were used in this experiment. All ewes were kept in feedlot pens and were fed Tifton hay and a balanced feed ration (150 g/ewe/day) with unlimited access to water and mineral salt. The ewes were synchronized (4 or 6 ewes/day) via the insertion (day -1) of a CIDR for 7 d. One day after CIDR insertion (day 0), half of the ewes were randomly divided to receive an i.m. injection of 0.5 mg of E-17β dissolved in 1 mL of sesame oil (Estradiol-17β; *n* = 31) or just an i.m. injection of sesame oil (Control group; *n* = 31). Ovarian superstimulation protocol (Brasil et al., 2016) was adapted to initiate FSH administration at follicular wave emergence. Therefore, 133 mg of pFSH i.m. (Folltropin®-V, Tecnopec, AHC Inc., Bioniche, Canada) in eight decreasing doses (20% x 2; 15% x 2; 10% x 2; 5% x 2) were administered twice a day, starting in the morning of day 3 and ending 12 hours after CIDR removal. All animals received an i.m. injection of 250 μg of cloprostenol (Sincrocio®, Ouro Fino Animal Health LTDA, Brazil) and 25 μg of gonadorelin acetate (Gestran Plus, Tecnopec, ARSA SRL, Argentina) together with 5th and 8th FSH injection, respectively. In addition, on day 3, the mean diameter of the largest follicle and the total number of antral follicles ≥ 1 mm were evaluated via transrectal ultrasonography in 12 ewes from each experimental group (Figure 1), randomly chosen. The following data were recorded: the mean size of the largest follicle; total number of small follicles (1 mm ≤ SF <4 mm); and the percentage of ewes with medium or large follicles (MF/LF ≥ 4 mm).

Three rams with proven fertility were used as semen donors for all AI. The rams were previously selected for the following seminal characteristics: (1) concentration ≥ 3 x 10^9^ mL^-1^; (2) motility mass ≥ 3; (3) sperm motility ≥ 80%; (4) vigor ≥ 3; and (5) total spermatic pathology ≤ 20%. All females were inseminated with fresh undiluted semen via cervical insemination. The AI was performed 36 and 48 h after CIDR withdrawal, using 0.5 mL straws. Immediately before AI, the semen of the rams was collected and pooled in a water bath at 33°C. The ewes were restrained with their hind limbs raised and the cervix was located using a speculum fitted with a light source. The semen was deposited as far as possible into the cervix without using force. Two days after the second AI, the previously used CIDR was reinserted into each female and kept in place until embryo collection (Figure 1).

On day 5 after the second AI, the number of ovulations was recorded using a laparoscopic procedure. Corpora lutea were classified as normal or prematurely regressed (PRCL), according to development status and color. Ewes with > 2 healthy CL were considered responsive donors. Immediately afterward, embryos were surgically recovered under general anesthesia.

After the ewes were deprived of food and water for 24 h, they were administered xylazine (0.10 mg/kg i.m.; Rompun®, Bayer, Brazil) and ketamine hydrochloride (3.5 mg/kg intravenous; Ketamina, Agener, Brazil). Furthermore, local anesthesia was administered on the location of the surgical incision (10 mL of lidocaine; Lidovet®, Bravet, Brazil). Ova/embryos were collected surgically after ventral laparotomy, using a cranial paramedian incision (5 cm long) to the udder to access the reproductive tract. Each uterine horn was flushed with 60 mL of embryo recovery medium (DPBS, Cultilab, Brazil), prewarmed to 38ºC, and supplemented with 1% fetal bovine serum (Cultilab, Brazil). Briefly, the flushing medium was injected using a sterile syringe with a 20G catheter inserted near the uterotubal junction and collected via a n.8 or n.10 Foley catheter that had been inserted at the external bifurcation of each uterine horn. Embryos were recovered in a Petri dish, maintained in holding media (Holding plus, 0.4% BSA, Embriocare, Cultilab, Brazil), and examined under a stereomicroscope (Olympus SZ; Olympus Optical Co., Ltd., Tokyo, Japan) at 20 to 40× magnification, pursuant to the International Embryo Transfer Society criteria. Embryos developing to the blastocyst or morula stage were graded as follows: grade 1 (excellent or good), 2 (good/fair), 3 (poor), and 4 (dead or degenerated). Embryos graded 1 to 3 were considered transferable and those were graded 1 and 2 were considered freezable embryos. After surgery, all ewes were given an i.m. injection of a luteolytic dose of PGF2α (250 µg cloprostenol) and the CIDR was removed.

The following data were recorded for each ewe: number of corpus luteum (CL), total recovered oocytes/embryos structures (TRS), viable embryos (VE), freezable embryos (FE), degenerated embryos (DGE), and total embryos (TE). The fertilization rate (FR) was obtained by dividing TE by TRS; the degenerated rate (DGER) was obtained by dividing DGE by TE; and recovery rate (RR) was obtained by dividing TRS by CL.

### Statistical analysis

Statistical tests were performed using the SAS University Edition program (SAS Institute Inc., Cary, NC, USA). In Experiment 1, ovarian follicular data were compared among four groups through the mixed generalized linear model (GLIMMIX procedure) and the mean of least squares were adjusted for multiple comparisons by the Royen-Tukey-Kramer test. The synchrony of the new follicular wave emergence was compared between the groups with F test (variance ratio) associated with the graphical analysis of the density distributions. In Experiment 2, ovarian responses and embryo yields were compared between treatments with or without E-17β using the GLIMMIX procedure and the mean of least squares were adjusted by the Royen-Tukey-Kramer test. Data recorded as percentages were analyzed by Fisher's exact test (donors with > 2 CL and donors with PRCL) and chi-square (degenerated embryos and fertilization). The number of small follicles (1 ≤ SF < 4mm) and the average diameter of the largest follicle evaluated by ultrasonography on day 3 were compared between the groups using Student's t test. In addition, Spearman correlations were performed between the number of small follicles at the beginning of FSH treatment and superovulatory response and embryo production of the donors. Differences were considered statistically significant at P ≤ 0.05. Results are expressed as the mean ± SD or in percentages.

## Results

### Experiment 1

The effect of P4 alone or combined at different doses of E-17β on follicular and luteal characteristics in cyclic hair sheep are shown in Table 1. The diameter of the largest follicle and CL at CIDR insert (day -1), as well as largest follicle diameter at E-17β administration (day 0) was similar among the groups tested (P > 0.05).

**Table 1:** Mean (± S.D.) follicular and luteal characteristics in cyclic hair sheep receiving a CIDR insert 1 d prior to treatment with different dosages of estradiol-17β (E-17β).

**Ovarian Status**	**Control**		**Estradiol-17β**		
	**0 mg**		**0.5 mg**	**1.0 mg**	**2.0 mg**
LF† at CIDR insert (mm)	4.7 ± 0.8		5.0 ± 1.5	4.9 ± 1.1	4.7 ± 0.6
CL at CIDR insert					
Number of ewes (%)	85.7 (6/7)		85.7 (6/7)	85.7 (6/7)	85.7 (6/7)
Diameter (mm)	10.0 ± 1.8		8.1 ± 1.7	7.8 ± 1.3	8.0 ± 1.2
LF at E-17β administration (mm)	4.7 ± 0.7		5.3 ± 1.4	4.7 ± 0.9	4.7 ± 0.7
**Follicular Wave Dynamics**					
Emergence (days)	0.86 ± 1.0^a^		2.8 ± 0.4^b^	3.2 ± 0.9^b^	3.4 ± 1.5^b^
Synchrony of follicular wave emergence					
Variance	0.98 ^b^		0.15 ^a^*	0.74 ^b^*	2.29 ^b^
Coefficient of variation (%)	116.3		14.3	28.1	44.1
Growth rate (mm/day)	0.9 ± 0.4		0.8 ± 0.3	0.9 ± 0.5	0.8 ± 0.4
Growth phase (day)	2.1 ± 0.6		2.4 ± 0.6	1.8 ± 0.9	1.9 ± 0.2
Static phase (day)	1.1 ± 0.4		1.4 ± 0.3	1.3 ± 0.3	1.4 ± 0.4
Wave length (day)	3.2 ± 0.8		3.7 ± 0.4	3.1 ± 1.1	3.3 ± 0.4
LF of wave (mm)	4.8 ± 0.6		4.7 ± 0.4	4.4 ± 0.6	4.4 ± 0.4

^a,b^ Different letters in the rows indicate significant differences (P <0.05). ^†^LF: largest follicle. *P: 0.079.

The number of days for follicular wave emergence did not differ among E-17β Groups (overall average = 3.1 ± 1.0). However, was significantly earlier in the control group (0.86 ± 1.0; P < 0.01). There was no difference in synchrony of follicular wave emergence among the control and 1.0 and 2.0 mg of E-17β groups (P > 0.05). However, administration of 0.5 mg E-17β induced a better synchrony of follicular wave emergence than control and 2.0 mg of E-17β groups (P < 0.05) and tended to be more synchronized than 1.0 mg of E-17β group (P = 0.079). The patterns of ovarian follicular development were similar (P > 0.05) among all groups (Table 1).

### Experiment 2

**Figure 2:**
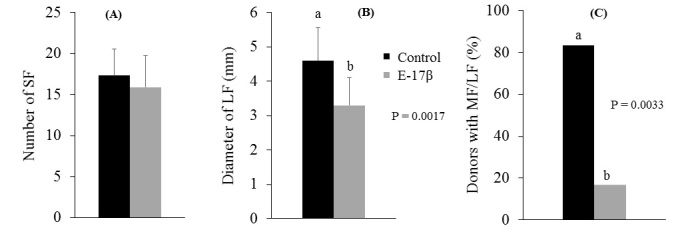
Follicular characteristics evaluated by ultrasonography on day 3 in ewes of the E-17β group (gray bars) and control group (black bars). (A) number of smaller follicles (1 mm ≤ SF ≤ 4 mm; mean ± SD); (B) median diameter of the largest follicle (mean ± SD); (C) donor ewes with medium/large follicles (MF/LF ≥ 4 mm; percentage). Within sets of data, bars with different lower cases letters show statistical differences (P < 0.01).

Follicular characteristics evaluated by ultrasonography on day 3 are shown in Figure 2. The number of small follicles at the moment of follicular wave emergence (day 3) did not differ between control and E-17β groups (17.3 ± 3.2 vs. 15.9 ± 3.8, respectively). However, the mean diameter of the largest follicle (4.6 ± 1.0 vs. 3.3 ± 0.8) and the percentage of ewes with medium/large size follicles (10/12, 83.3% vs. 2/12, 16.7%) were significantly lower in ewes treated with E-17β (P < 0.01).

The effects of E-17β treatment on superovulatory response and embryo yield in cyclic hair sheep are shown in Table 2. There were no significant differences between the two groups of ewes for any of the embryo yield variables analyzed (P > 0.05). The number of small follicles in the first administration of FSH was not correlated with ovulatory response (0.15; P = 0.51) and viable embryos (0.09; P = 0.69).

**Table 2:** Ovarian response and embryo production (Mean ± S.D.) after multiple doses of FSH treatment in cyclic hair sheep treated and not treated with 0.5 mg of E-17β, 24 h after CIDR insertion.

**Variables**	**Estradiol-17β**	**Control**	***P value***
Donors with > 2 CL (%)	25/31 (81)	27/31 (87)	0.73
Donors with ERCL (%)	5/31 (16)	10/31 (32)	0.24
Number of CL	9.6 ± 4.7	11.2 ± 6.9	0.70
Total recovered structures	7.7 ± 4.7	7.3 ± 4.7	0.78
Total embryos	6.2 ± 4.4	6.1 ± 4.6	0.87
Viable embryos	4.8 ± 4.3	4.7 ± 4.3	0.90
Freezable embryos	4.0 ± 4.0	4.2 ± 4.3	0.84
Degenerated embryos rate (%)	34/153 (22)	36/164 (22)	0.95
Fertilization rate (%)	153/193 (79)	164/198 (83)	0.37

ERCL: Early regressed corpora lutea.

## Discussion

The combination of progesterone with 0.5 mg of E-17β it seems to improve the synchrony of the emergence of a new follicular wave. Moreover, in the second experiment, the administration 0.5 mg of E-17β allowed superovulation treatment started in the appearance of a follicular wave, but does not improve superovulatory response and embryo production.

Our results showed that the interval between treatment and wave emergence did not differ between the three dosages of E-17β, with an overall mean of 3.1 d after E-17β treatments. However, the combination of progesterone with E-17β delayed the follicular wave emergence in relation to P4 alone (Control). Similar to our results, studies using administration of 5.0 mg of E-17β combined with progesterone at random stages of follicular growth in heifers caused the synchronized emergence of a new follicular wave within 2 d (Garcia and Salaheddine, 2001) to 3.4 d (Martínez et al., 2000). In anestrous ewes, a lower dose than that used in this study (0.35 mg of E-17β and MAP) caused the emergence of a new wave later, after about 5 d (Barrett et al., 2008), and in cyclic ewes did not synchronize the emergence of new follicular wave. It is known that after the end of the blockage of gonadotrophin concentrations caused by progesterone in combination with E2, FSH concentrations increased again, reaching the peak preceding the emergence of the follicular wave (Bo et al., 1995; Bo et al., 1994). It is likely that the suppression of FSH release caused by E2 is lower in cyclic ewes, which would require larger doses, such as those used in this experiment, to cause atresia of the largest follicle and emergence of a new follicular wave.

In the different groups, a similar pattern of ovarian follicular development after administration of E-17β was demonstrated, with the exception that the synchrony of the emergence of the new wave. The 0.5 mg dose improved the synchrony of the emergence of the new wave with compared to 2.0 mg and tended to improve in 1.0 mg dose. The ewes treated with 0.5 mg of E-17β and progesterone (Experiment 1), the emergence of new wave occurred between 2.5 and 3.5 d. While dosages of 1.0 and 2.0 mg of E-17β produced a variation of between 2 and 4.5 d and 2 and 6 d, respectively. The reason the group that received the dose of 0.5 was more synchronized is not clear. However, lack of synchrony and delay in the emergence of the follicular wave may result from extended high concentrations of estradiol, such as those observed when using estradiol which has a long half-life (Garcia and Salaheddine, 2001). This is an important finding due to a growing concern with reducing hormone protocols used in animal breeding.

The results of our study showed that on the day expected for follicular emergence, i.e., day 3 following the administration of 0.5 mg of E-17β, the number of small follicles was similar to that of the control group, which were at random phase follicular growth. It is probable that the number of small follicles present in the ovary is more dependent upon intrinsic factors in each female (Mossa et al., 2007) than on the follicular growth phase. Furthermore, it was intriguing to note that there was no correlation between the amount of these small follicles and the superovulatory response and embryo production, contrary to previous studies (González-Bulnes et al., 2000; Mossa et al., 2007; Bartlewski et al., 2008b). It is possible that there is not very accurate in the use of ultrasound to identify follicles with a size close to 1 mm, leading to contradictory results.

It is known that, in bovines, there is an increase in the number of small follicles at the emergence of the follicular wave, whereas the establishment of dominance reduces the follicular population present in the ovaries (Bungartz and Niemann, 1994; Ginther et al., 1996; Ferreira et al., 2004). In contrast, in cyclic ewes, the number of small follicles (1 to 3 mm in diameter) does not change throughout the estrus cycle, with no evidence of the effect of follicular dominance on the number of these follicles (Duggavathi et al., 2003).

Several studies support the idea that the superstimulation treatment initiated in the absence of a dominant follicle in sheep results in an increase in the embryonic yield (González-Bulnes et al., 2002; Veiga-Lopez et al., 2005; Rubianes et al., 1995; Veiga-Lopez et al., 2008b; Menchaca et al., 2010). In the present study, ewes treated with 0.5 mg E-17β presented a lower follicles size at the beginning of ovarian superstimulation with FSH (3.3 ± 0.8 vs. 4.6 ± 1.0; P < 0.01, Figure 2). However, contrary to expectations, there was no improvement in ovulation rate and embryonic yield. A probable suppressive effect of the larger follicle on the other follicles of the same wave was observed in the ultrasound evaluations (Veiga-Lopez et al., 2005; Veiga-Lopez et al., 2008a). However, it is believed that this effect is minor, since it is not uncommon for more than one follicle to reach preovulatory size and for more than one follicle to ovulate in the same wave. Furthermore, it is physiologically possible that the ovulation of follicles in multiple waves may occur (Seekallu et al., 2010; Toosi et al., 2010). In sheep submitted to a unilateral ovariectomy there is an increase in ovulation of the follicles of the penultimate wave, with a compensatory effect on the rate of ovulation (Duggavathi et al., 2008). All this leads us to believe that the presence of a large follicle at the beginning of the ovarian superstimulation treatment has little or no effect on the final embryo yield.

Early regressed corpora lutea (ERCL) is an inherent problem in ovine superovulation, which usually results in poor quality embryo recovery or even no recovery (Cognie, 1999; Schiewe et al., 1991). In an attempt to avoid this problem, all donor ewes in this experiment had a second-use CIDR reintroduced after AI. However, this approach did not prevent the appearance of ERCL in both groups (16% and 32% in the E-17β and control group, respectively).

The nutritional management in each experiment was differentiated to meet the nutritional requirements of the animals. In experiment 1, the animals had access to pasture, while in experiment 2 (dry period) they were confined and supplemented with balanced commercial concentrate. All ewes were kept in good body condition in both experiments. In this way, we believe in a minimal effect of the feeding management of animals on the results of the experiments, according to previous studies (Naqvi et al., 2002; Kakar et al., 2005). In addition, the treatments performed were contemporary in each experiment.

Superovulatory protocols initiated at or around the time of follicle wave emergence in cattle (Bo et al., 2002; Mapletoft et al., 2002) and in the absence of large follicles in ewes (Rubianes et al., 1995; González-Bulnes et al., 2002) improves embryonic yield. However, we demonstrated that superovulatory protocols initiated at the time of emergence of the follicular wave does not improve superovulatory response and embryo production in hair sheep. We demonstrated that there were no differences in the number of small follicles at the beginning of the superovulation treatment, even though in the control group there was a greater number of ewes with large follicles.

## Conclusion

It is concluded that P4 in combination with 0.5 mg E-17β was more efficient at synchronizing follicular wave emergence in ewes. However, the pFSH superstimulation initiated at the predicted time for follicular wave emergence does not improve superovulatory response and embryo production.
